# *In vivo* nanoparticle-mediated radiopharmaceutical-excited fluorescence molecular imaging

**DOI:** 10.1038/ncomms8560

**Published:** 2015-06-30

**Authors:** Zhenhua Hu, Yawei Qu, Kun Wang, Xiaojun Zhang, Jiali Zha, Tianming Song, Chengpeng Bao, Haixiao Liu, Zhongliang Wang, Jing Wang, Zhongyu Liu, Haifeng Liu, Jie Tian

**Affiliations:** 1 Key Laboratory of Molecular Imaging, Institute of Automation, Chinese Academy of Sciences, Beijing 100190, China; 2 Beijing Key Laboratory of Molecular Imaging, Beijing 100190, China; 3 Department of Gastroenterology, General Hospital of Chinese People's Armed Police Forces, Beijing, 100039, China; 4 Department of Nuclear Medicine, Chinese PLA General Hospital, Beijing, 100853 China; 5 School of Life Science and Technology, Xidian University, Xi'an 710071, China; 6 Department of Nuclear Medicine, Xijing Hospital, Fourth Military Medical University, Xi'an 710032, China; 7 Anal-colorectal Surgery Institute, No. 150 Central Hospital of PLA, Luoyang 471031, China; 8 The State Key Laboratory of Management and Control for Complex Systems, Chinese Academy of Sciences, Beijing 100190, China

## Abstract

Cerenkov luminescence imaging utilizes visible photons emitted from radiopharmaceuticals to achieve *in vivo* optical molecular-derived signals. Since Cerenkov radiation is weak, non-optimum for tissue penetration and continuous regardless of biological interactions, it is challenging to detect this signal with a diagnostic dose. Therefore, it is challenging to achieve useful activated optical imaging for the acquisition of direct molecular information. Here we introduce a novel imaging strategy, which converts γ and Cerenkov radiation from radioisotopes into fluorescence through europium oxide nanoparticles. After a series of imaging studies, we demonstrate that this approach provides strong optical signals with high signal-to-background ratios, an ideal tissue penetration spectrum and activatable imaging ability. In comparison with present imaging techniques, it detects tumour lesions with low radioactive tracer uptake or small tumour lesions more effectively. We believe it will facilitate the development of nuclear and optical molecular imaging for new, highly sensitive imaging applications.

## Abstract (web summary)

Insufficient imaging sensitivity can make it challenging to assess early stage tumour lesions *in vivo*. Here, the authors present the radiopharmaceutical-excited fluorescence imaging technique that can detect tumour lesions less than 2 mm in size in living mice.

## Graphical abstract

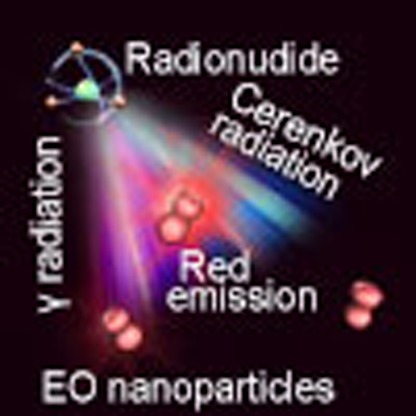

I*n vivo* Cerenkov luminescence imaging (CLI) is an emerging technique that can be used to visualize the biological distribution of radiopharmaceuticals through Cerenkov luminescence (CL)^1^,^2^,^3^,^4^,^5^,^6^,^7^,^8^,^9^,^10^,^11^,^12^,^13^. Similar to bioluminescence and fluorescence molecular imaging (BLI and FMI), CLI offers high surface resolution and high throughput^13^,^14^. Unlike positron emission and single-photon emission computed tomography (PET and SPECT), CLI can image both β^−^ and β^+^ particle emitting isotopes^11^,^15^. Since many clinically approved nuclear tracers produce Cerenkov light, CLI shows great promise for clinical translation and has been recently applied for human thyroid and lymphatic node imaging^16^,^17^.

Despite these achievements, CLI faces many challenges. Cerenkov luminescence is weak with an intensity that is several orders of magnitude less than that of standard fluorescence imaging^17^,^18^. This is because most of the radiated energy is in the form of γ photons and/or other emitted particles generated during radioactive decay. The CL spectrum is weighted towards ultraviolet and blue^15^,^19^, which leads to high absorption in biological tissues and limits the imaging depth. Like γ radiation in nuclear imaging, CL is not ‘triggered' light but is continuously generated during radioactive decay regardless of biological interactions. To achieve ‘activatable' imaging of nuclear agents, whose optical signal is activated on relevant biological interactions, the fluorescent conversion of CL has been proposed^19^,^20^,^21^,^22^,^23^. However, the overall optical signal must be killed for energy transformation, which limits imaging sensitivity.

To overcome these challenges, especially, to achieve activatable imaging and enhance the optical signal, we present a novel *in vivo* imaging strategy, radiopharmaceutical-excited fluorescence imaging (REFI). It utilizes europium oxide (EO) nanoparticles to convert γ-radiation (major) and CL (minor) into fluorescence. This is similar to a process called radioluminescence, which uses ionizing radiation (X- or γ-radiation) to irradiate nanophosphors and generate light. Phosphorous materials have been used in radiation detectors for many years. A recent publication reported measurements of Eu(II) and Eu(III) in the new CsSrI3:Eu scintillator using X-ray absorption spectroscopy^24^. However, adopting this concept for bioimaging is quite recent and underexplored^25^,^26^,^27^,^28^. There is no comparison between this approach and other imaging techniques for highly sensitive *in vivo* tumour detection yet. Our technique combines the merits of CLI and FMI to boost the light intensity and signal-to-background ratio and achieve internal activatable imaging via the excitation from two different electromagnetic radiations. To investigate the excitation mechanisms, the signal enhancement, the emission spectrum, the tissue penetration ability and the difference in outcomes between REFI, CLI, FMI and PET, a series of *in vitro* phantom and *in vivo* xenograft studies are conducted. The benefits of REFI for highly sensitive tumour imaging are demonstrated in two scenarios. We believe that by combining the strengths of CLI and FMI, signal-enhanced optical imaging through internal radionuclide excitation with dual electromagnetic radiations will benefit preclinical research and clinical diagnosis in the future.

## Results

### EO morphology and spectrophotometry

**Figure 1:**
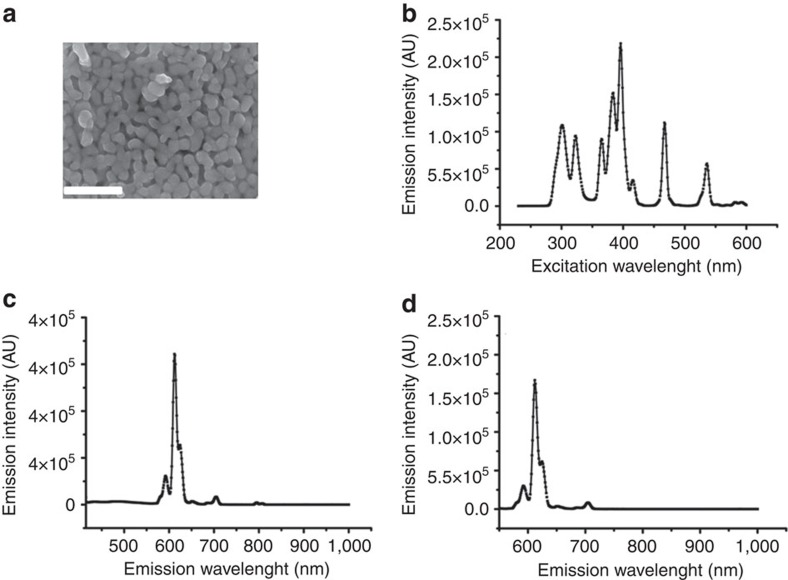
EO nanoparticle morphology and fluorescence characterization. (**a**) SEM visualization of EO nanoparticles with mean size of 85 nm. Scale bar, 500 nm. (**b**) The optical excitation spectrum with a 620 nm filter displaying the characteristic absorption peaks of EO at 301, 323, 365, 384, 396, 467 and 536 nm. (**c**,**d**) The emission spectra of EO excited by 400- and 535-nm lasers displaying the peak emission with both excitation wavelengths at 620 nm.

Scanning electron microscopy (SEM) showed EO nanoparticles with peanut-like morphology (Fig. 1a). The average diameter of EO nanoparticles was 85±22 nm. The ζ potential was 28.6 mV. The excitation profile is shown in Fig. 1b. There were multiple characteristic absorption peaks at 301, 323, 365, 384, 396, 467 and 536 nm. The emitted fluorescence spectra of EO excited by 400- and 535-nm lasers are shown in Fig. 1c,d. The peak emission was 620 nm in both cases. The absolute quantum yield of EO was 39%.

### The mechanism of radiopharmaceutical excitation

The radiopharmaceutical imaging tracer ^18^F-FDG (fluorodeoxyglucose) emits γ-radiation (511 keV), β^+^ particles and CL, whereas ^99m^Tc-MDP (methylene diphosphonate) only emits γ-radiation (140 keV). Therefore, we employed both radioactive tracers to reveal the mechanism of radioactive tracer-excited fluorescence from EO.

**Figure 2:**
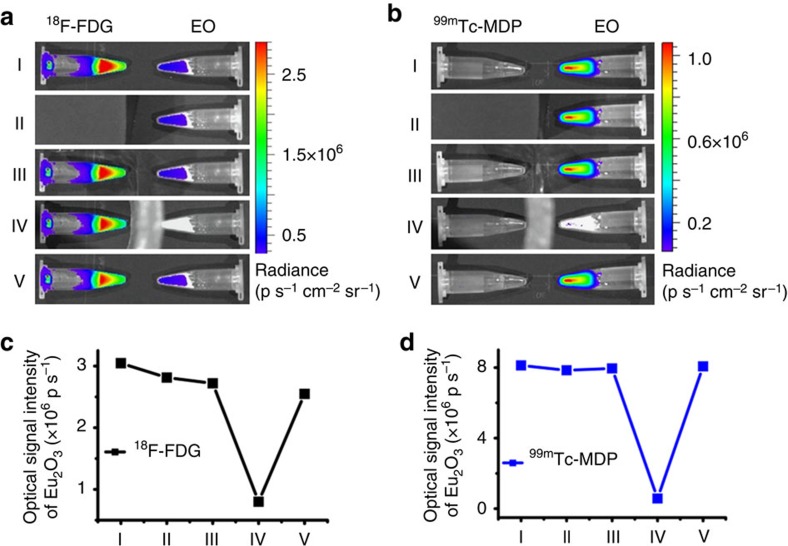
The study of the radiopharmaceutical excitation mechanism. (**a**,**b**) EO is excited by ^18^F-FDG (**a**) and ^99m^Tc-MDP (**b**) with different radiation blocking conditions. From row I to V: normal view, black box blocking CL, aluminium foil blocking β^+^, lead torus blocking γ, normal view. (**c**,**d**) The emission intensity of each condition is plotted for using ^18^F-FDG (**c**) and ^99m^Tc-MDP (**d**), respectively. Both cases reveal obvious intensity decrease in Condition IV. The slight intensity decrease from Condition I to V in (**c**) indicates the relatively shorter half life of ^18^F.

Our previous study illustrated that an excitation range from ultraviolet to blue can excite EO to generate red emission (620 nm). This indicated that CL (350–450 nm weighted) was also capable of exciting EO. However, it was not known whether it was the only radiation from radionuclides causing the excitation. Figure 2 demonstrates that neither blocking Cerenkov light (Row II) nor blocking β^+^ radiation (Row III) significantly influenced the induction of fluorescence from EO. Conversely, the fluorescent signal decreased dramatically when the γ radiation from ^18^F-FDG or ^99m^Tc-MDP was blocked (Row IV). Therefore, γ radiation was the major cause of EO excitation. Both high (511 keV) and low energy (140 keV) γ-rays were able to achieve EO excitation.

**Figure 3:**
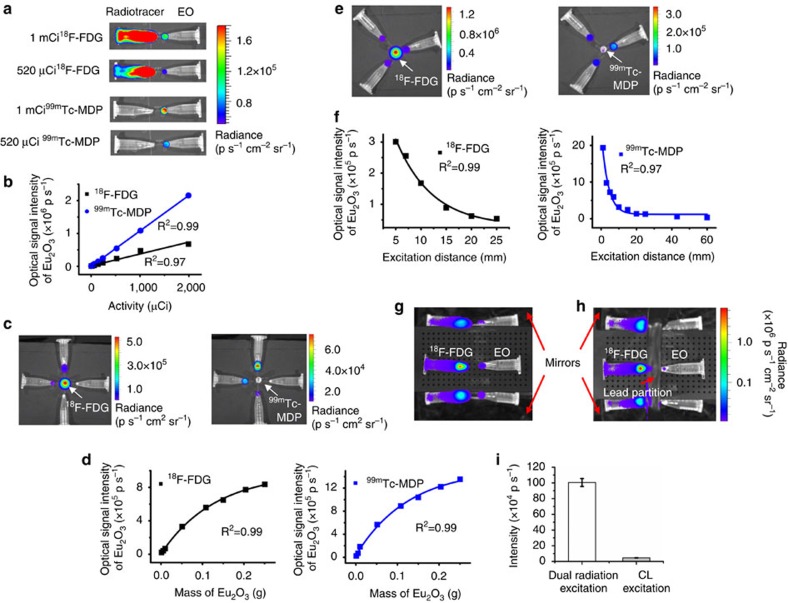
The investigation of different factors influencing the excitation. (**a**) The emission intensity of EO depends on the radioactivity and the radiotracers. (**b**) The linear relationship between emission intensity and radioactivity. ^18^F-FDG and ^99m^Tc-MDP shows different excitation efficiencies. (**c**) The emission intensity of EO depends on its mass. (**d**) The exponential relationship between emission intensity and EO mass. (**e**) The emission intensity of EO depends on the excitation distance. (**f**) The inverse relationship between emission intensity and excitation distance. (**g**,**h**) Using mirrors and a lead partition to achieve γ and CL dual excitation (**g**) and pure CL excitation (**h**), respectively, to measure the fractions of γ- and Cerenkov excitation. (**i**) The quantification reveals that CL contributed a small portion of the dual-radiation excitation.

The fluorescent emission of EO increased linearly with increasing activity of radioactive tracers (Fig. 3a,b). The excitation efficiency of ^99m^Tc-MDP was better than that of ^18^F-FDG. Fluorescent emission strongly correlated with tracer activity with *R*^2^ values of 0.97 (^18^F-FDG) and 0.99 (^99m^Tc-MDP). For both ^18^F-FDG and ^99m^Tc-MDP excitation, the emitted fluorescent intensity of EO increased exponentially with an increase in its mass (Fig. 3c,d). Fluorescent emission strongly correlated with the amount of EO with an *R*^2^ value of 0.99. With increasing excitation distance, the EO emission intensity decreased exponentially (Fig. 3e,f). Fluorescent emission strongly and inversely correlated with distance with *R*^2^ values of 0.99 for ^18^F-FDG excitation and 0.97 for ^99m^Tc-MDP excitation. The fluorescent signal was almost undetectable when the excitation distance between ^18^F-FDG and EO was over 25 mm.

The fractions of γ- and Cerenkov excitation in REFI are dependent on radioactivity, γ- photon energy, EO mass, and excitation distance. For a single study (100 μCi ^18^F-FDG + 10 mg EO with 10 mm distance), we employed a lead partition and two sets of mirrors (Fig. 3g,h) to obtain dual-radiation excitation and CL radiation excitation separately. The induced mean optical signal of EO was (100.5±5) × 10^4^ p s^−1^ and (4.6±0.1) × 10^4^ p s^−1^, respectively (Fig. 3i). Therefore, the fractions of γ- and CL excitation were 95.4 and 4.6% in this case, which proved γ-radiation was the major source of excitation.

### Different excitation efficiencies

**Figure 4:**
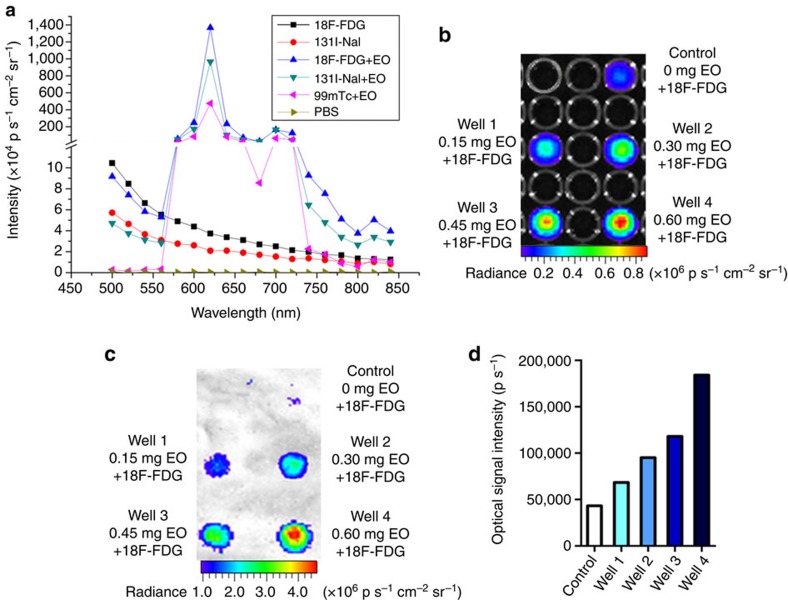
The emission profiles and the tissue penetration assessment. (**a**) The emission spectra of ^18^F-FDG, ^18^F-FDG+EO, ^131^I-NaI, ^131^I-NaI+EO, ^99m^Tc-MDP+EO and PBS (each radiotracer, 140 μCi; EO, 10 mg). The spectra of ^18^F-FDG and ^131^I-NaI indicate the Cerenkov light is weighted towards blue (< 500 nm), and the spectra of ^18^F-FDG+EO, ^131^I-NaI+EO and ^99m^Tc-MDP+EO show a similar profile with peak emission at 620 nm. The EO absorbance of part of the Cerenkov light is indicated by the difference between the emission spectra of radiotracer with and without EO from the wavelength of 500–570 nm. (**b**,**c**) The comparison between Cerenkov luminescence (Control, 50 μCi ^18^F-FDG) and ^18^F-FDG radiation-excited fluorescence (well 1–4) before (**b**) and after (**c**) covering with a piece of 1-mm thick porcine gastric mucosa tissue. (**d**) The quantification of the optical intensity of each well after covering the tissue.

Figure 3b indicates that different radioisotopes with the same activity caused different excitation efficiencies, and ^99m^Tc-MDP with lower γ photon energy (140 keV) was better than ^18^F-FDG (511 keV), when the radiotracer was separated with EO for excitation. However, when we co-mixed different radiotracers, 140 μCi ^18^F-FDG, ^99m^Tc-MDP and ^131^I-NaI (^131^I-sodium iodide, major γ energy 364 keV), with 10 mg EO, respectively, and investigate their emission spectra, we found the opposite (Fig. 4a). ^18^F-FDG+EO showed the highest signal, and ^99m^Tc-MDP+EO was the lowest. It was a surprise that ^18^F and ^99m^Tc showed different relative excitation efficiency in conditions of separation and mixture. We hypothesized that the photoelectric interactions and Compton scattering of europium and γ-photons were related to the distance between radionuclide and europium. When mixing the ^18^F with EO, both β^+^ particles and γ-photons transferred energy to extranuclear electrons of EO and resulted in visible photons. However, ^99m^Tc only emits γ photons, thus its excitation efficiency was lower than ^18^F in case of mixing with EO. When radiotracer was separated from EO for certain distance, the probability of β^+^ particles reaching EO was almost zero. It was γ photons that dominated the excitation for both isotopes. However, the probability of Compton scattering for 511 keV γ photon might be higher than that for 140 keV γ photon, which did not induce optical signal. The probability of photoelectric effect for 140 keV γ-photon might be higher than that for 511 keV γ-photon, which induced optical signal. That was the reason why separating and mixing radiotracers with EO showed different excitation efficiency.

To further explore the relationship between emission intensity and excitation photon energy below 140 keV, we built a hybrid X-ray and optical system (Supplementary Fig. 1a). X-ray with the energy range of 40 to 130 keV was used to excite the EO (Supplementary Fig. 1b), and the emission intensity increased linearly with an *R*^2^ value of 0.95 (Supplementary Fig. 1c).

### Optical signal enhancement and spectral red shift

With the presence of 10 mg EO, the optical signal enhancement showed a marked effect (Fig. 4a). The emission intensity was two orders of magnitude more than the normal Cerenkov luminescence, even with the only 2-min exposure (Note that it took 5-min exposure time for the rest of our studies of REFI and CLI.). This together with our previous mechanism studies (Fig. 2) proved that EO was utilized to achieve fluorescent conversion of γ-radiation emitted from radiopharmaceuticals to boost the overall optical signal. Besides that, all three combinations (^18^F-FDG, ^99m^Tc-MDP or ^131^I-NaI+EO) showed similar spectra between 570 and 740 nm. The peak of the emission profile was at 620 nm followed by a smaller peak at 700 nm. It is notable that for wavelength <570 nm, the overall optical signal was weaker than Cerenkov signal emitted from ^18^F or ^131^I along. This indicated that the blue Cerenkov light also contributed the excitation of EO, therefore part of its energy was shifted from blue to red. The radiopharmaceutical induced fluorescence showed greater signal intensity and shifted the optical spectrum from blue towards red. Both improvements suggested better ability to penetrate tissues. Since the emission profiles were similar for all three radiotracers, and the most clinically used ^18^F-FDG demonstrated the best excitation efficiency in the case of co-mixing EO, we focused on the ^18^F-FDG with EO for the rest of our studies.

Figure 4b,c shows the difference in tissue penetration ability between CLI and REFI. The Cerenkov luminescent signal of the control well (50 μCi ^18^F-FDG) was nearly ablated (from 6.4 to 0.4 × 10^5^ p s^−1^) after covering with a piece of porcine gastric mucosa tissue (1-mm thick). However, the REFI signal was greater in Wells 1 to 4, which contained a range of amounts of EO (0.15–0.60 mg) mixed with ^18^F-FDG (50 μCi each). The signal intensity of Well 4 (50 μCi ^18^F-FDG + 0.6 mg EO) was approximately fourfold greater (1.8 × 10^5^ p s^−1^) than that of the control (Fig. 4d).

### Combining the merits of CLI and FMI

**Figure 5:**
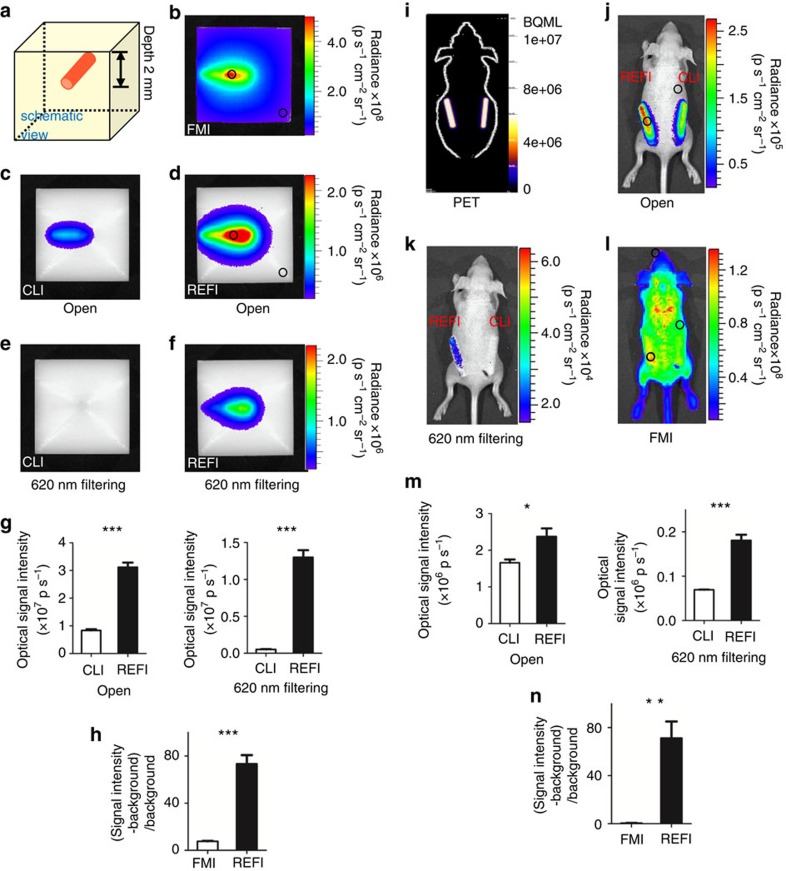
The *in vitro* and *in vivo* phantom comparison between different imaging techniques. (**a**) A schematic illustration of the *in vitro* tissue-mimicking phantom. (**b**) FMI of the 100 μCi ^18^F-FDG+1 mg EO mixture. (**c**,**d**) With no filtering, CLI using ^18^F-FDG only (**c**) and REFI using the mixture of EO and ^18^F-FDG (**d**) show obvious different signal intensity. (**e**,**f**) With 620-nm filtering, CLI (**e**) and REFI (**f**) also demonstrate intensity differences. (**g**) The optical intensity comparison between CLI and REFI with no filtering and 620-nm filtering. (**h**) The signal-to-background ratio comparison between FMI and REFI. (**i**) The PET image of the *in vivo* phantom shows no significant difference between the two implanted glass tubes (left tube: 50 μCi of ^18^F-FDG+0.15 mg of EO mixture, right tube: ^18^F-FDG, 50 μCi). (**j**,**k**) REFI and CLI show significant differences with no filtering (**j**) or 620-nm filtering (**k**). (**l**) FMI of the *in vivo* phantom. (**m**) The quantitative comparison of CLI and REFI in optical intensity with no filtering and 620 nm filtering. (**n**) The comparison of FMI and REFI in signal-to-background ratio.

CLI does not need external excitation as FMI does, which excludes background reflection and autofluorescence. Thus, an improved signal-to-background ratio is one of the strengths of CLI. REFI relies on radiation from a radiopharmaceutical to excite EO internally. Combining REFI with CLI confers the absence of external excitation and gives REFI the merit of a high signal-to-background. In both *in vitro* and *in vivo* phantom studies, the signal-to-background ratio of REFI was significantly larger than that of FMI (*in vitro P*<0.001, *in vivo P*<0.01, Fig. 5). Regions of interest are shown with black circles, and quantifications are listed in Fig. 5 and Supplementary Table 1.

The activatable imaging strategy of external excitation in FMI is advantageous in terms of molecular specificity. Conversely, radioactive probes are constantly emitting CL during decay regardless of their interactions with biological molecules. This excludes CLI from the benefits of biologically specific, activatable imaging. However, REFI incorporates γ-radiation and CL to excite EO internally for fluorescent emission. This strategy conferred the merit of FMI for activatable imaging to REFI. For *in vitro* phantom imaging (Fig. 5a–h), the activated REFI signal was distinguished from conventional CLI by either optical signal enhancement (Fig. 5c,d, *P*<0.001) or 620-nm filtering (Fig. 5e,f, *P*<0.001). For *in vivo* phantom imaging (Fig. 5i–n), PET showed no significant differences (*P*>0.05) between the two implanted capillary tubes (Fig. 5i), which indicated that the two tubes were filled with the same dose of ^18^F-FDG (50 μCi). However, the signal intensity was significantly different between CLI and REFI with no filtering (Fig. 4j, *P*<0.05) or filtering (Fig. 4k, *P*<0.001).

### *In vivo* validation of the superiorities of REFI

**Figure 6:**
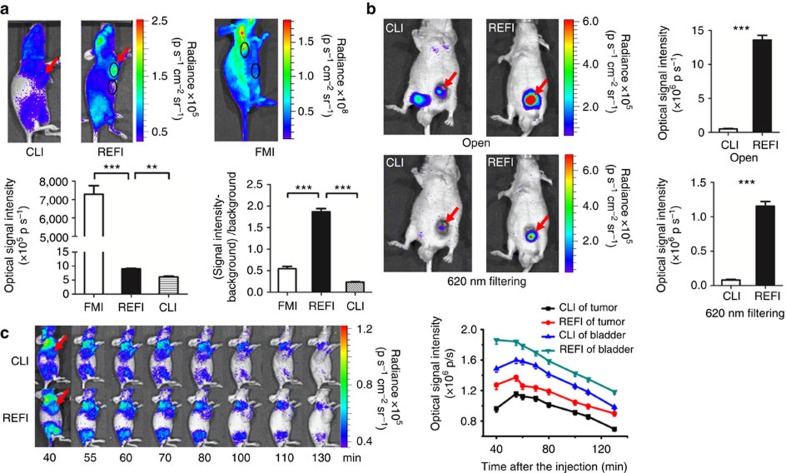
The comparison between different optical techniques for *in vivo* imaging xenografts. (**a**) After direct intratumoural injection with EO (0.05 mg) and tail-vein injection of ^18^F-FDG (800 μCi) into the Bcap-37 xenografts, REFI shows the best tumour to normal tissue contrast among all three imaging modalities. (**b**) After tail-vein injection with EO (0.1 mg, 24 h prior) and ^18^F-FDG (500 μCi) in the U87MG-xenografted mice, REFI shows significantly greater signal than CLI did with both no filtering and 620-nm filtering. This demonstrates the passive accumulation of the EO nanoparticle in the tumour tissue. (**c**) The longitudinal observation comparing CLI and REFI in Bcap-37 xenografts. EO (0.1 mg) and 480 μCi of ^18^F-FDG are mixed and injected via the tail-vein. Both tumour and bladder show greater optical signal compared with that in the control mice at all time points.

EO nanoparticles were directly injected into the tumours of Bcap-37 (human breast cancer cell) xenograft mice to simulate tumour-targeted nanoparticle delivery. Ten hours later, ^18^F-FDG was tail-vein injected. Figure 6a shows a comparison of REFI, CLI, and FMI *in vivo*. All three imaging techniques successfully detected optical signals from tumours. However, REFI exhibited both high optical intensity (REFI versus CLI: 8.43±1.35 versus 3.69±0.84, unit: 10^5^ p s^−1^, *P*<0.01) and high signal-to-background ratio (REFI versus FMI: 1.74±0.17 versus 0.94±0.14, *P*<0.01). Therefore, REFI offered the best tumour to normal tissue contrast in all cases. The superiorities of REFI were consistent with the results of the previous phantom studies. We also performed a comparison between REFI and CLI, in which EO was tumour injected after the tail-vein injection of ^18^F-FDG. The signal enhancement effect was significant too (*P*<0.01, Supplementary Fig. 2).

REFI and CLI were compared using U87MG (human gliomablastoma cell) and Bcap-37 xenograft mice models to verify the passive accumulation of EO nanoparticles in tumours via enhanced permeability and retention (EPR) effect after intravenous (i.v.) injection.

For *in vivo* imaging of U87MG xenografts, REFI with no filtering or 620-nm filtering showed significantly stronger optical signal (both cases *P*<0.001) compared with that of CLI (Fig. 6b). The mean intensity of REFI (no filtering: 135.7±12.1 × 10^5^ p s^−1^, 620 nm filtering: 115.3±11.6 × 10^4^ p s^−1^) was about 27 times and 15 times greater than that of CLI (no filtering: 5.1±1.6 × 10^5^ p s^−1^, 620-nm filtering: 7.9±2.1 × 10^4^ p s^−1^) with no filtering and 620 nm filtering, respectively.

For *in vivo* longitudinal imaging of Bcap-37 xenografts, the consistent phenomenon was observed. For both tumour and bladder, the optical signal of REFI was significantly stronger than that of CLI at all time points (Fig. 6c). This indicated that EO particles may partially excrete through kidney and urinary system. However, the intensity differences were not as great as in the U87MG xenograft experiment. This may have been because the injection of EO was performed 24 h earlier than the injection of ^18^F-FDG in the U87MG group, whereas the EO and ^18^F-FDG were injected together in the Bcap-37 group. This may have resulted in further passive accumulation of EO inside U87MG tumour tissues.

We also performed the comparison between FMI using ICG (indocyanine green) and REFI using ^11^C-CHO (Choline) with EO through HepG2 (human hepatic cancer cell) orthotopic liver tumour mice models. The results further proved that REFI offered much better tumour to normal tissue contrast than FMI did (*P* < 0.01, Supplementary Fig. 3).

### Multimodality comparison for tumour detection

**Figure 7:**
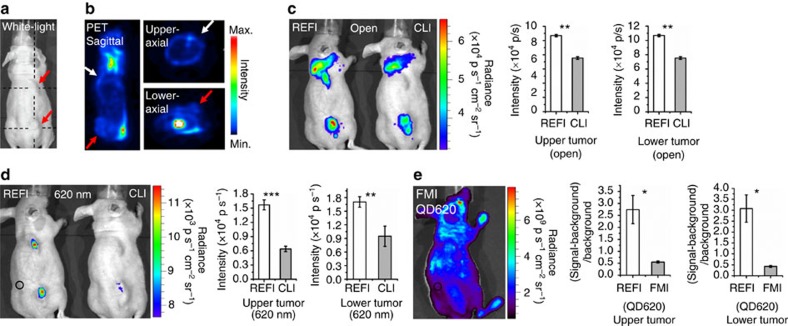
The multimodality comparison for *in vivo* imaging the dual-tumour xenografts. (**a**) Six days after the subcutaneous injection of 4T1-luc2 tumour cells, two tumour lesions are visible on the back of a mouse model (red arrows). (**b**) Three slices of PET (280 μCi ^18^F-FDG) in sagittal and axial directions show clear ^18^F-FDG uptake in the lower tumour (red arrows) but no significant uptake in the upper one (white arrows). The position of the three PET slices are indicated in (**a**) with black dotted lines. (**c**) Without filtering, REFI (280 μCi ^18^F-FDG with 0.1 ml, 1 mg ml^−1^ EO) shows optical signal of both tumours and brown adipose tissue (left mouse), but CLI does not visualize the upper back tumour (right mouse). (**d**) With 620 nm filtering, two tumours are visualized in REFI (left mouse), but Cerenkov signal are nearly vanished. (**e**) FMI of QD620 (0.1 ml, 10 mg ml^−1^) does not show great tumour to normal tissue contrast due to the non-specificity of the fluorescent probe. The black circle indicates the regions of interest (ROI) of the background for calculating signal-to-background ratio.

**Figure 8:**
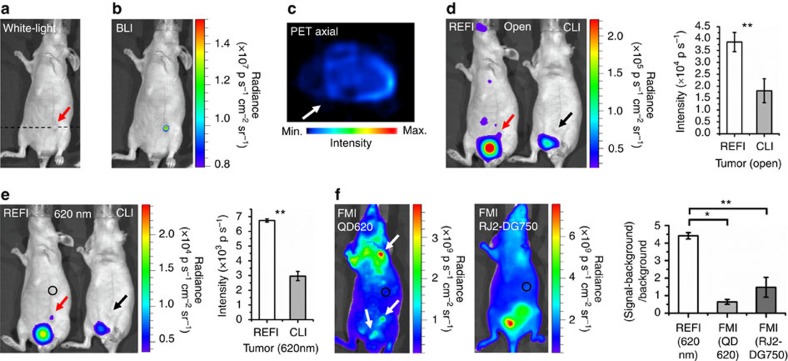
The multimodality comparison for *in vivo* early detection of small tumour lesions. (**a**) Sixty-five hours after tumour cell injection, a white light image shows a small tumour lesion (red arrow). (**b**) BLI confirms the location of the small tumour lesion. (**c**) Axial PET shows false-negative scan. (**d**,**e**) With no filtering and 620 nm filtering, CLI (right mouse) shows false-negative detection (black arrows), but REFI (left mouse) shows true-positive detection (red arrows). (**f**) FMI of untargetted QD620 shows multiple suspected lesions, and targeted RJ2-DG750 shows overestimation of the tumour region. The signal-to-background ratios of both fluorescent probes are significant lower than that of REFI.

To investigate the advantages of REFI for sensitive imaging of tumour lesions, 4T1-luc2 (luciferase-expressing mouse adenocarcinoma cell) xenografts were used for multimodality imaging comparison. PET, CLI, FMI and REFI were applied for two scenarios, and REFI offered the best imaging performance in both cases. The same dose of ^18^F-FDG was i.v. injected into the xenografts for PET, CLI and REFI. All quantifications of optical intensity and signal-to-background ratio are listed in Figs 7 and 8, and Supplementary Table 2.

Six days after subcutaneous injection of 4T1-luc2 tumour cells, the tumour lesions were clearly visible on the upper and lower back of each mouse (Fig. 7a) with average diameter of 5.6±0.8 mm. PET can detect the lower back tumour (Fig. 7b red arrows), yet cannot detect the upper back one (Fig. 7b white arrows). This was because the brown adipose tissue (high ^18^F-FDG uptake) close to the upper tumour influenced its uptake of the radiotracer. CLI confirmed this phenomenon (Fig. 7c, right mouse). Optical signal were detected from brown adipose tissue and lower tumour lesion, but upper tumour was still not visualized. However, with the presence of EO and ^18^F-FDG in the same tumour lesion, the internal radiopharmaceutical excitation was activated. Significant optical signal enhancement (REFI versus CLI: upper tumour: *P*<0.01, lower tumour: *P*<0.01) was detected in both tumours for REFI (Fig. 7c, left mouse). After 620-nm filtering, most of the Cerenkov luminescence was blocked (Fig. 7d, right mouse), including the optical signal from the brown adipose tissue (Fig. 7d, left and right mice). However, REFI offered significant optical signal for upper (REFI versus CLI: *P*<0.001) and lower (REFI versus CLI: *P*<0.05) tumour lesions (Fig. 7d, left mouse).

In previous phantom imaging and *in vivo* imaging studies, the FMI showed relatively worse performance partially due to the low quantum yield of EO. To make the comparison fair and thorough, the quantum dot 620 (QD620) was used in this study. It is ‘untargeted', same as EO, but with absolute quantum yield of more than 80% (more than twice of EO). Besides that the dose of QD620 (0.1 ml, 10 mg ml^−1^) was 10 times higher than the dose of EO (0.1 ml, 1 mg ml^−1^) for *in vivo* imaging. However, REFI still demonstrated significant better signal-to-background ratio with the same excitation and emission setting up (REFI versus FMI: upper tumour: 2.74±0.59 versus 0.55±0.02, *P*<0.05, lower tumour: 3.07±0.63 versus 0.43±0.05, *P*<0.05, Fig. 7e).

Sixty-five hours after subcutaneous injection of 4T1-luc2 tumour cells, the tumour size was only 2.1±0.3 mm (Fig. 8a, red arrow). BLI visualized the tumour lesion (Fig. 8b), but PET offered a negative scan (Fig. 8c). This insufficient sensitivity of PET is probably due to the partial volume effects that caused significant underestimation of radiotracer concentration in small lesions^29^,^30^,^31^. CLI confirmed this again (Fig. 8d,e, black arrows). It was really difficult to detect the week Cerenkov signal from such a small lesion. However, REFI successfully achieved a positive imaging in both no filtering and 620-nm filtering modes (Fig. 8d,e red arrows, REFI versus CLI: both *P*<0.01).

To make a comprehensive comparison between FMI and REFI, we employed two fluorescent probes this time, the untargeted QD620 and targeted RediJect 2-DeoxyGlucosone 750 (RJ2-DG750). RJ2-DG750 is a probe for targeting of tumours that exhibit elevated glucose uptake rate in comparison with surrounding tissues. The FMI of QD620 showed multiple suspected lesions (Fig. 8f, white arrows), whereas the FMI of RJ2-DG750 showed an overestimated tumour lesion area (Fig. 8f, right mouse). Nevertheless, the signal-to-background ratios of FMI were still significantly smaller than that of REFI (REFI versus FMI: 4.42±0.18 versus 0.64±0.14 (QD620), *P*<0.05, 4.42±0.18 versus 1.48±0.57 (RJ2-DG750), *P*<0.01).

To further validate above findings that REFI was more sensitive than PET for small tumour detection, another *in vitro* phantom and *in vivo* breast cancer mice model studies were conducted (Supplementary Figs 4 and 5). The results also proved that REFI was indeed more sensitive in small tumour detection.

### Biodistribution of EO and toxicity evaluation

**Figure 9:**
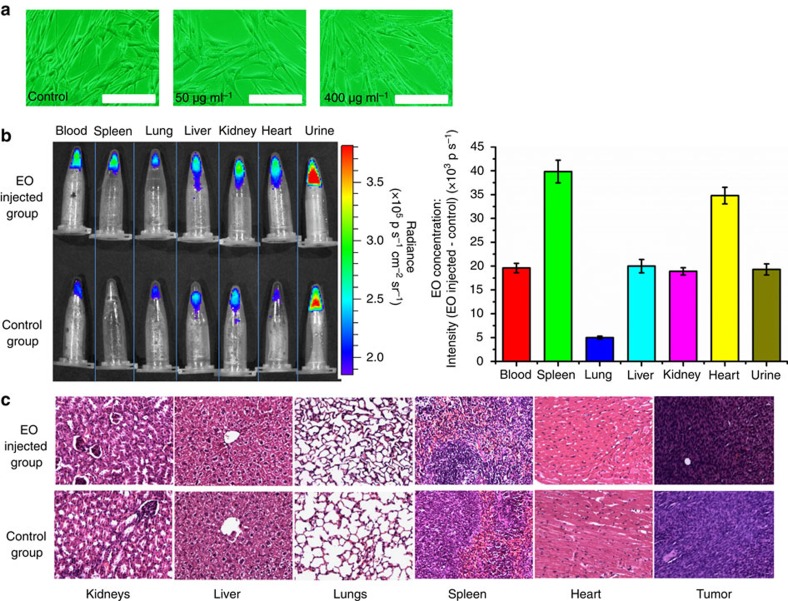
Biodistrubition of EO and toxicity evaluation using MTT assays and H&E microscopy. (**a**) *In vitro* cytotoxicity assay indicated no significant cell morphologic changes and no obvious cell aggregation. Scale bar, 200 μm. (**b**) The optical signal difference of the EO-injected group and control group (left image, upper row and bottom row) indicated the different concentrations of EO in tissues, blood and urine samples. The quantitative measurements showed that liver, kidneys and urine had a similar EO concentration, which proved the reason of signal enhancement in the bladder during *in vivo* REFI imaging. The high EO concentration in the spleen was probably because of phagocytosis by macrophages. (**c**) Compared with the control tissues, the kidneys, liver, lungs, spleen, heart and tumour of the EO tail-vein-injected group (Bcap-37 xenografts) did not show obvious structural changes.

Cytotoxicity assay of EO was performed. Human umbilical cord mesenchymal stem cell morphology after 24 h of incubation with 50 and 400 μg ml^−1^ EO was captured using fluorescence microscopy (Fig. 9a). Neither significant change of cell morphology nor cell aggregation was observed in the experimental sample compared with that of the control sample (Fig. 9a) without nanoparticles.

The biodistribution of EO was measured 40 min after the tail-vein injection of EO (0.1 ml, 1 mg ml^−1^). Vital organs, such as heart, kidney, liver, lung and spleen, were ground using tissue-grinding pestles. Each sample (0.1 ml), including the sample of blood and urine, was mixed with ^18^F-FDG (0.1 ml, 100 μCi) respectively, so that the REFI signal was induced. By comparing the optical intensity with the control samples obtained from mice without the injection of EO (pure CL signal), the signal difference reflected the biodistribution of EO per 0.1 ml (EO concentration). The results showed the spleen and heart had the highest EO concentration. Blood, liver, kidney and urine were very similar, and lung had the lowest EO concentration (Fig. 9b). This proved that EO was partially accumulated in liver, and partially excreted in urine through kidney.

Haematoxylin and eosin (H&E) microscopy (Fig. 9c) showed no obvious structural change in kidneys, lungs, spleen, liver, heart or tumour of the experiment group with EO injection. Compared with QD620, the EO nanoparticles were much less toxic, as all mice injected with QD620 died within the next 4 h, but all mice injected with EO lived more than 4 weeks except killed mice.

## Discussion

This study establishes REFI as a novel imaging strategy. Using EO nanoparticles and clinical radiopharmaceuticals, the γ- (major) and Cerenkov (minor) radiation can be converted to fluorescence to achieve internally excited optical imaging. Our series of *in vitro* studies identified the excitation source and illustrated the relationship between the emission intensity and various parameters, such as radioactivity, EO mass and excitation distance. The excitation efficiency of different radiotracers and X-ray photon energies was investigated. The optical signal enhancement, spectra red shift and signal tissue penetration were also demonstrated.

REFI utilized both γ- and Cerenkov radiation from radioactive tracers to achieve internal activatable imaging. This unique feature eliminates the adverse effects of autofluorescence and reflection from external excitation as occurs in conventional fluorescence molecular imaging and enhances emission signals through fluorescent conversion of γ-radiation to overcome the challenge of detecting a weak Cerenkov signal from radioisotopes. Comparison of REFI, CLI and FMI through phantom *in vitro* and Bcap-37, U87MG and 4T1-luc2 xenograft *in vivo* studies clearly demonstrated that REFI combined the merits of CLI and FMI with superb signal-to-background ratio and internal activatable imaging ability. Especially in the 4T1-luc2 xenograft study of small tumour detection, REFI showed significant better signal-to-background ratio than FMI did, no matter it was QD620 (higher quantum yield) or RJ2-DG750 (better targeting specificity) that was applied in FMI. Furthermore, in comparison of CLI, the optical signal of REFI was boosted remarkably and was spectrally shifted towards the deeper tissue-penetrating red range. All these features benefited the optical imaging performance in our *in vitro* and *in vivo* studies.

REFI employed both radiotracer and EO nanoparticles, and the internal signal activation highly relies on the distance of them. Therefore, if tumour lesion shows similar uptake of one tracer but higher uptake of the other, in comparison with surrounding normal tissues, the tumour to normal tissue contrast will stand out. Our dual tumour 4T1-luc2 xenograft study demonstrated this phenomenon through multimodality comparison of REFI, PET and CLI. The uptake of ^18^F-FDG in upper back tumour was influenced by brown adipose tissue nearby, but REFI was able to visualize the tumour unaffectedly, whereas PET and CLI failed.

The sensitivity of PET is superior to many other imaging modalities, but it is still limited by the low resolution partially due to the partial volume effects^29^,^30^,^31^. For small tumour lesion with the size smaller or close to its spatial resolution, the underestimation of radiotracer uptake becomes significant. The high superficial resolution of optical imaging, the signal enhancement effect and the better signal-to-background ratio empowered REFI to detect small subcutaneous tumour lesions more effectively. Our 4T1-luc2 xenograft study indicated that REFI was able to detect tumour lesions with the size <2 mm, which were <3 days after tumour transplanting in nude mice. This suggested a great potential of applying REFI for highly sensitive early tumour detection and tumour metastasis imaging, even with passive delivery of the nanoparticle. Different from bioluminescence imaging, REFI does not require incorporation of immunogenic proteins^32^,^33^, such as luciferase and GFP (green fluorescent protein). It thus can be applied to a wider range of animal tumour models and holds better clinical translation potential.

Other studies of Cerenkov-induced fluorescence imaging (SCIFI) have already demonstrated the mechanism of utilizing Cerenkov light solely for excitation and applications of SCIFI in tumour marker detection using targetted fluorescent probes^19^,^22^. The fundamental difference between REFI and SCIFI (or other similar approaches) is the utilization of γ-radiation for optical excitation and the resulted signal enhancement, as the overall optical signal of SCIFI was even weaker than Cerenkov light because of the inevitable energy loss during excitation. There were also studies of using radioluminescent nanoparticles and radiotracers, such as ^18^F-FDG, for biomedical imaging applications^23^,^25^,^26^,^27^, but these works remained in phantom studies and did not reveal the dual-radiation excited imaging mechanism. To the best of our knowledge, this is the first *in vivo* small animal tumour model study using REFI or other similar approaches.

The EO nanoparticle is not the only choice for applying the strategy of REFI. The lanthanide-doped nanoparticle is likely to provide similar fluorescence conversion of both γ- and Cerenkov radiation from radioisotopes^26^,^27^. These can be excited by collimated X-rays (approximately the same energy magnitude as γ-radiation from ^99m^Tc-MDP) and have already been applied in X-ray/optical imaging modalities, such as X-ray luminescence computed tomography^27^,^34^,^35^. Therefore, our imaging technique converts external high-energy (X-ray) and low-energy (optical light) electromagnetic excitation to internal γ- and CL excitation via radiopharmaceuticals for *in vivo* optical molecular imaging.

We believe that REFI and its principle hold great promise for *in vivo* activatable imaging that can detect molecular targets or events and provide quantitative information of pathological processes on molecular levels with proper modification of the EO or other lanthanide-doped nanoparticle to overcome the issue of target specificity. REFI, with its intrinsic superiorities, will benefit the imaging of tumour-to-tumour molecular heterogeneity, which can further facilitate the development of precision medicine and personalized patient care. Therefore, this imaging technique will expand the applicability of activatable nuclear and optical molecular imaging.

In conclusion, through utilizing EO nanoparticles mediators, we have achieved internal conversion of continuous radiopharmaceutical radiation to activatable fluorescence for molecular imaging. REFI can merge the advantages of nuclear and optical molecular imaging techniques *in vivo*. This creates a strong motivation for further modification of the EO nanoparticle to obtain biomarker specificity and application of this highly sensitive imaging technique to early and small tumour detection in the near future.

## Methods

### Nanoparticles and radionuclide tracers

The EO nanoparticle (Eu2O3, 99.9% metal basis, molecular weight=351.91) was purchased from the Aladdin Chemistry Co. Ltd. ^18^F-FDG, ^99m^Tc-MDP and ^131^I-NaI was provided by the department of nuclear medicine, Chinese PLA General Hospital, Beijing, China. The QD620 was purchased from China Beijing Beida Jubang Science & Technology Co. Ltd, and the RJ2-DG750 probe was purchased from PerkinElmer. The absolute quantum yield of EO and QD620 was measured using FLS980 fluorescence spectrometer (Edinburgh Instruments). Both kinds of nanoparticle were dissolved in PBS for measurements.

### SEM and spectrophotometry

The size and morphology of EO nanoparticles were determined by SEM (Hitachi S-4700). As-prepared EO powder samples were dispersed and dropped on a copper grid for scanning. The particle size was quantified using Image J. The EO nanoparticles were characterized for fluorescent properties using an EnSpire Multimode Plate Readers (PerkinElmer). The samples were read using a transparent 96-well plate. The excitation profile was obtained using a 620-nm emission filtre. The fluorescence profiles were obtained with excitation at 400 and 535 nm.

### Mechanistic study of radiopharmaceutical excitation

The imaging system used for these studies was the IVIS Spectrum system (Caliper Life Sciences). The imaging parameters were binning: 4, exposure: 5 min and aperture: f1, unless otherwise indicated.

In the identification of the excitation source experiment, two 1.5-ml tubes filled with 0.2 g EO and 600 μCi of ^18^F-FDG were placed 15 mm apart inside the imaging chamber and a sequence of images were acquired. The first image was taken in the normal view. The second was taken with a black cardboard box covering the ^18^F-FDG tube. The third and fourth images were taken with an aluminium foil and a lead torus placed between the two tubes, respectively. Finally, a normal view of the two tubes was acquired again. Images of tubes with 990 μCi of ^99m^Tc-MDP and 0.20 g of EO were acquired following the same protocol.

In the emission intensity versus radioactivity experiment, EO (10 mg) was excited using 0.1 ml of ^18^F-FDG and 0.1 ml of ^99m^Tc-MDP with 11 different activities (2, 5, 10, 19, 30, 63, 140, 240, 521, 1,010 and 1,990 μCi). The images were analysed quantitatively to obtain the relationship between the emission intensity and radioactivity. The excitation distance was 10 mm.

In the emission intensity versus EO mass experiment, ^18^F-FDG and ^99m^Tc-MDP of 100 μCi of were used to excite EO in eight different amounts (0.0012, 0.0052, 0.0099, 0.0511, 0.1082, 0.1501, 0.2042 and 0.2505, g). The excitation distance was 10 mm.

In the emission intensity versus excitation distance experiment, the excitation distance was set to 1, 3, 5, 7, 10, 15, 20, 25, 43 and 60 mm, respectively. The radioactivity of ^18^F-FDG and ^99m^Tc-MDP was 250 μCi in each acquisition, and 10 mg of EO was used.

For the assessment of γ and Cerenkov excitation fraction, a pair of mirrors was set next to the two EP tubes containing ^18^F-FDG (100 μCi) and EO (10 mg), respectively. The tips of two EP tubes were 12 mm apart. After taking optical images, a lead partition (12-mm thick) was put in between the EP tubes to block γ-radiations from ^18^F. However, the CL was partially reflected to the EO because of the two mirrors reflection. Then optical images were taken again for pure CL excitation.

### Emission spectra assessment

^18^F-FDG, ^99m^Tc-MDP and ^131^I-NaI (each 140 μCi) were mixed with EO nanoparticles (10 mg), respectively. Their emission spectra together with the Cerenkov luminescent spectra of ^18^F and ^131^I (both 140 μCi) were measured using the IVIS system (exposure: 2 min). PBS was used as the Control.

### X-ray excitation of EO nanoparticles

During the X-ray excitation process, the voltage of the X-ray tube (Hamamatsu L9181-02 Microfocus X-ray Source, Japan) was increased gradually, while the tube current was kept constant. Therefore, X-ray photons with different emission energies (40, 60, 80, 100, 110, 120 and 130 keV) were obtained to excite EO nanoparticles (2 mg) inside an EP tube (Supplementary Fig. 1b), but the total photon number of the X-ray beam was kept constant for each excitation. To protect the EMCCD (DU888+, Andor, UK), the optical imaging system was perpendicular to the X-ray tube (Supplementary Fig. 1a). The white light image was acquired with a 0.1-s exposure inside room light, and the X-ray-excited fluorescent image was acquired with 5-s exposure inside a light sealed environment. The PBS was used as control.

### Excitation efficiency of different radiopharmaceuticals

^18^F-FDG, ^99m^Tc-MDP and ^131^I-NaI (each 100 μCi) were used to excite 10 mg EO, respectively (mixing excitation). The optical images were acquired using the IVIS Spectrum system (Caliper Life Sciences) with binning: 4, exposure: 2 min and aperture: f1. The excitation efficiencies of using different radiotracers were compared (Supplementary Fig. 1d).

### Biological tissue penetration assessment

The biological tissue penetration ability of the fluorescence emission was evaluated using a piece of 1-mm thick porcine gastric mucosa tissue dissected from a freshly butchered porcine stomach. ^18^F-FDG of 50 μCi was injected into one well as the control. The other four wells were injected with the mixture of ^18^F-FDG (50 μCi) and EO (0.15, 0.30, 0.45 or 0.60 mg). Images without and with tissue blocking were taken (binning: 8, exposure: 2 min).

### Comparison of optical imaging techniques via phantoms

Two identical tissue-mimicking phantoms were made from high-density polyethylene to simulate the optical properties of mouse muscle^36^. Each was a cube that was 40 × 40 × 40 mm^3^ in dimension. A small hole (diameter 2.5 mm, length 20 mm) was drilled 2 mm beneath the surface (Fig. 4a). A 0.1-ml mixture of ^18^F-FDG (100 μCi) and EO (1 mg) or 0.1 ml ^18^F-FDG (100 μCi) alone was injected into each hole. CLI, REFI and FMI were performed for comparison. For CLI and REFI, both 620-nm filtered and unfiltered images were acquired (exposure 5 min). For FMI, the excitation and emission wavelengths were 465 and 620 nm (exposure 1 s). The experiment was performed in triplicate.

Two glass tubes were filled with 0.1 ml of ^18^F-FDG (50 μCi) or a 0.1-ml mixture of ^18^F-FDG (50 μCi) and EO (0.15 mg). They were then subcutaneously implanted into a euthanized nude mouse. PET (Genisys PET, SofieBiosciences), CLI, REFI and FMI were performed. The experiment was performed in triplicate.

### Animal experiments

All animal experiments were conducted in compliance with the guidelines of the Institutional Animal Care and Use Committee of General Hospital of Chinese People's Armed Police Forces. All animal procedures were performed isoflurane gas anaesthesia (3% isoflurane–air mixture), and all efforts were made to minimize suffering. The Balb/c nude mice were obtained from the Laboratory Animal Center of the Chinese Academy of Medical Sciences. Seven- to eight-week-old mice were used for experiments. Female mice were applied for Bcap-37 and 4T1-luc2 studies, and male mice were applied for U87MG and HepG2 studies. The subcutaneous tumour and breast cancer mice models were established by subcutaneously injecting 5 × 10^6^ tumour cells in nude mice. The orthotopic liver tumour mice models were established by performing a laparotomy in mice under isoflurane gas anaesthesia and injecting 5 × 10^6^ HepG2 cells into the liver.

### *In vivo* validation of REFI

In the experiments of the intratumoural administration route, the control and experimental groups (three Bcap-37 xenografts per group) were tail-vein injected with 800 μCi of ^18^F-FDG. The tumour tissue of the experimental group received direct injection of 0.05 ml of EO solution (diluted in normal saline, 1 mg ml^−1^) 10 h prior to tail-vein injection. Forty minutes after the injection of ^18^F-FDG, CLI and REFI were performed for both groups (exposure 5 min) and the FMI was performed for the experiment group (exposure 2 s).

^18^F-FDG (100 μCi) was tail-vein injected into three 4T1-luc2 xenografts. CLI was performed 40 min after the injection (binning 4, exposure 5 min and aperture f1). Then, the EO solution (25 μl, 1 mg ml^−1^) was locally injected into the tumour lesions, and REFI was taken immediately (Supplementary Fig. 2).

In the experiments of i.v. administration, EO solution (0.1 ml, 1 mg ml^−1^) was tail-vein injected into three U87MG-xenografted mice as the experiment group. Twenty-four hours later, both control and experimental groups received injections of 0.1 ml of ^18^F-FDG (500 μCi). After another 40 min, CLI and REFI were conducted with 620-nm filtering and no filtering (exposure 5 min).

EO solution (0.1 ml, 1 mg ml^−1^) and 0.1 ml of ^18^F-FDG (480 μCi) were mixed and tail-vein injected into three Bcap-37 xenografted mice. The control group only received the injection of ^18^F-FDG (480 μCi). Longitudinal observations were performed at 40, 55, 60, 70, 80, 100, 110 and 130 min after the injection (exposure 5 min).

### *In vivo* comparison between REFI and FMI using ICG

Three HepG2 liver tumour mice models were used to compare the FMI and REFI. Volume of 0.1 ml ICG solution (1 mg ml^−1^) was tail-vein injected into the mice. Twenty-four hours later, EO (0.1 ml, 1 mg ml^−1^) and ^11^C-CHO (250 μCi) was tail-vein injected into the same mice. Forty minutes later, the abdominal cavity of the mice was surgically opened to expose the liver, and REFI was performed with binning 4, exposure 5 min, 620-nm filtering (Supplementary Fig. 3b). Then, FMI was performed with excitation 795 nm and emission 835 nm (binning 4, exposure 2 s, Supplementary Fig. 3a).

### *In vitro* phantom comparison between REFI and PET

A glass bottle was filled with 12 ml ^18^F-FDG with the concentration of 3.75 μCi ml^−1^. Then, a glass capillary 1 mm in diameter was inserted inside. The tip of the capillary was filled with 0.5 μl EO (1 mg ml^−1^) and 0.5 μl ^18^F-FDG (4.17 μCi ml^−1^). The capillary tip was used to simulate a very small tumour lesion with a volume of 1 mm^3^, and the glass bottle was used to simulate a mouse body. The small lesion had a slight higher uptake of ^18^F-FDG and EO than its surroundings. Then, the phantom was imaged using REFI (binning 4, exposure 5 min, no filtering) and PET respectively (Supplementary Fig. 4).

### *In vivo* multimodality comparison

EO solution (0.1 ml, 1 mg ml^−1^) and 0.1 ml of ^18^F-FDG (280 μCi) were mixed and tail-vein injected into three 4T1-luc2 dual tumour mice models. The mice received subcutaneous injection of 4T1-luc2 tumour cells 6 days ago. Volume of 0.1 ml of ^18^F-FDG (280 μCi) was injected into another three identical models. For each mouse, PET was scanned 35 min after injection with 10 min data acquisition, and then REFI and CLI were immediately performed with no filtering and 620 nm filtering (exposure 5 min). For FMI, three identical models received i.v. injection of QD620 (0.1 ml, 10 mg ml^−1^) and were scanned 50 min later, using system with 465 nm excitation and 620 nm emission (binning, 4 and exposure, 2 s).

In the experiments of *in vivo* small tumour lesion imaging, for PET, CLI and REFI, the protocols were exactly the same as the protocols of the dual tumour mice models imaging study, except that all tumour mice models were imaged 65 h after subcutaneous injection of 4T1-luc2 cells into the left lower abdominal mammary fat pad. For FMI, same protocol was applied for using QD620. However, for targetted fluorescence imaging, 0.1 ml (100 nmol ml^−1^) RJ2-DG750 was i.v. injected into the mice models. The mice were scanned 2 h later with 745-nm excitation and 800-nm emission (binning, 4 and exposure, 2 s).

Six 4T1-luc2 orthotopic breast cancer mice models were established to perform the comparison. Thirty-six hours after tumour transplantation, BLI was applied to verify the location of the tumour. Then PET, CLI and REFI were performed to detect the tumour. Sixty-five hours after tumour transplantation, the same procedure was applied again (Supplementary Fig. 5). For PET, CLI and REFI, the tail-vein injection of 100 μCi ^18^F-FDG was applied. For REFI (three of the six mice models), the tail-vein injection of 0.1 ml EO (1 mg ml^−1^) was applied.

### Cytotoxicity assay

*In vitro* cytotoxicity was measured by performing MTT assays on human umbilical cord mesenchymal stem cells. Different concentrations of EO (0, 50, 200 and 400 μg ml^−1^, diluted in PBS) were added to the wells. The samples were observed 24 h later using a conventional fluorescence microscope (Olympus IX71).

### Biodistribution of EO

Three mice were killed 40 min after the tail-vein injection of EO as the experiment group, and another three mice without EO injection were killed as control. Heart, kidney, liver, lung and spleen were ground using tissue-grinding pestles, and 0.2 ml PBS was added during grinding for dilution. Then each sample (0.1 ml), including 0.1 ml blood and 0.1 ml urine, was mixed with 0.1 ml ^18^F-FDG (100 μCi), respectively. Samples from the control followed the same procedure. Then optical images were acquired using the IVIS Spectrum system. The EO concentration in each sample was defined as optical signal intensity (EO-injected—control).

### Hematoxylin and eosin staining

To evaluate the tissue toxicity of EO, the Bcap-37 xenografted mice were killed immediately after *in vivo* imaging for histological examination. Tumours and organs were fixed in 4% formalin- and paraffin-embedded sections (4-μm thickness) were prepared for H&E staining. The slices were examined using a digital microscope (Leica QWin).

### Statistical analyses

Statistical comparisons were made using Student's *t*-test and GraphPad Prism 5 software. *P* values <0.05 were considered to indicate significance. Average and s.d. were calculated for experiments performed in triplicate. No s.d. indicated relevant measurement performed only once.

## Additional information

**How to cite this article:** Hu, Z. *et al.* *In vivo* nanoparticle-mediated radiopharmaceutical-excited fluorescence molecular imaging. *Nat. Commun.* 6:7560 doi: 10.1038/ncomms8560 (2015).

## Supplementary Material

Supplementary Information Supplementary Figures 1-5 and Supplementary Tables 1-2
