# Supplementary material for: In vivo nanoparticle-mediated radiopharmaceutical-excited fluorescence molecular imaging
Source: Nat Commun. 2015 Jun 30;6:7560. doi: 10.1038/ncomms8560 (PMC4491820; doi:10.1038/ncomms8560)
Supplement: Supplementary Information — Supplementary Figures 1-5 and Supplementary Tables 1-2 [file ncomms8560-s1.pdf]

Supplementary Information

Supplementary Figures

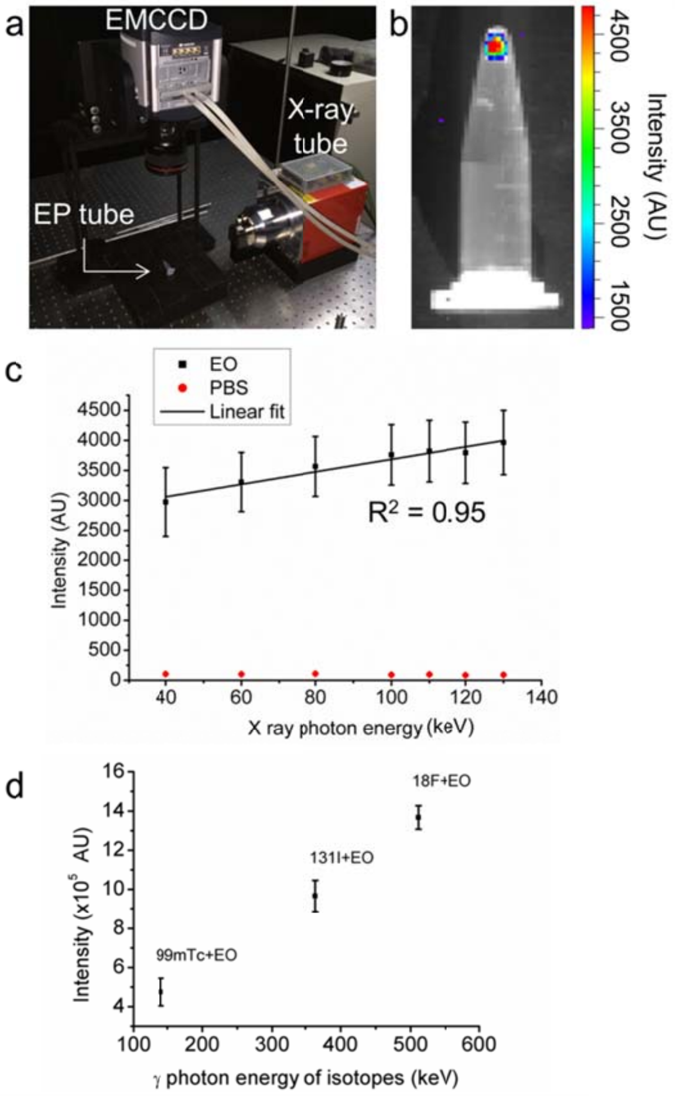

Supplementary Figure 1. Excitation efficiency assessment of X-ray and γ photons with different energy levels. (a) The hybrid X-ray and optical system. The EMCCD was water-cooled to -80 degrees Celsius to acquire X-ray excited fluorescent images. (b) The overlap of white light and fluorescent images (X-ray photon energy: 130 keV). (c) The linear relationship between optical signal intensity and X-ray photon energy. (d) The comparison of excitation efficiency for using different radiopharmaceuticals. ^{18}F-FDG (511 keV γ photons) showed the best efficiency.

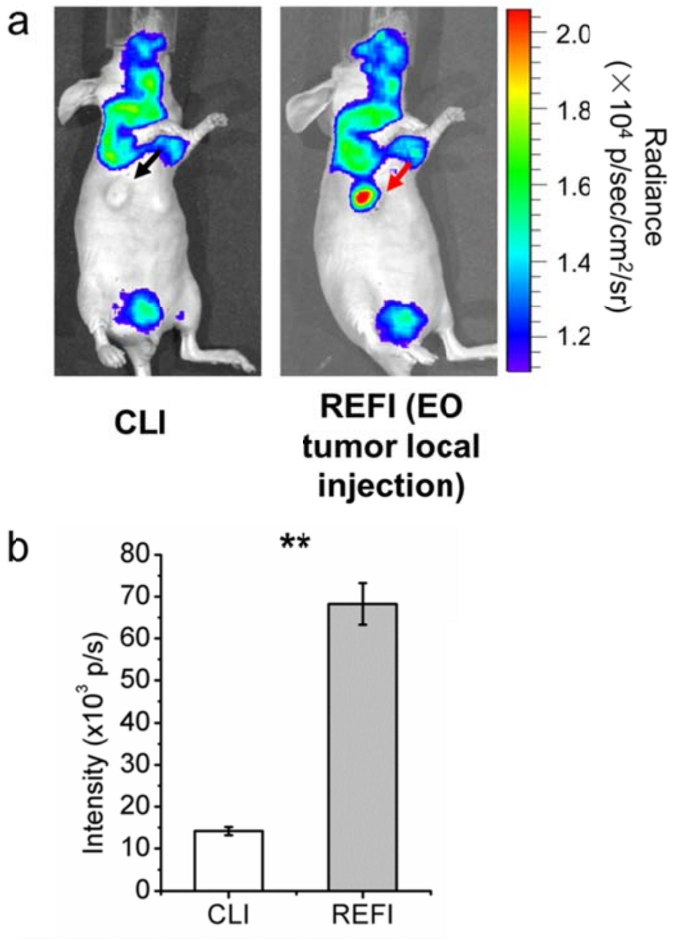

Supplementary Figure 2. The *in vivo* comparison between CLI and REFI using local tumor injection of EO. (a) 40 min after the tail vein injection of ^{18}F-FDG (100 µCi), CLI did not show an obvious optical signal from the tumor lesion (black arrow) due to the low dose of the radiotracer. However, after the tumor local injection of EO, REFI demonstrated a significant signal enhancement effect (red arrow). The signal from the tumor lesion was even stronger than the signal from the bladder. (b) The quantitative measurements of signal intensity. REFI (68.2 ± 5.0, unit: 10^3 p/s) was 4.8 times as large as CLI (14.1 ± 1.0, unit: 10^3 p/s).

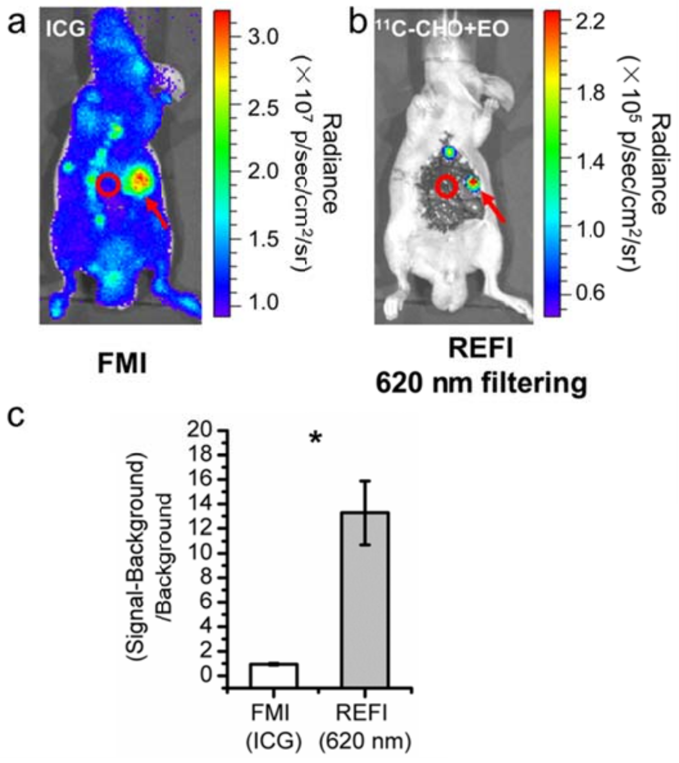

Supplementary Figure 3. The comparison between FMI using ICG and REFI using ^{11}C-CHO with EO. (a) FMI indicated the location of the liver tumor lesion (red arrow). (b) REFI with 620 nm filtering indicated the same location of the tumor lesion as FMI did (red arrow). (c) The quantitative calculation showed that the signal-to-background ratio of REFI was about 13.3 times more than that of FMI ($P < 0.05$). The ROI of the background was indicated by red circles in both (a) and (b).

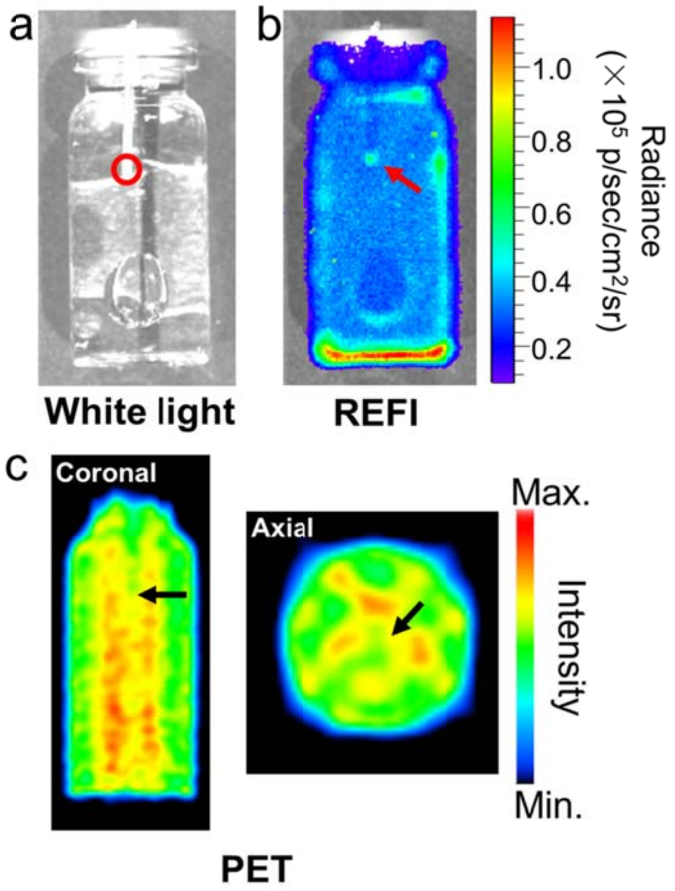

Supplementary Figure 4. The comparison between REFI and PET using a phantom. (a) The white light photography of the phantom. A glass bottle was filled with 12 ml ^{18}F-FDG (3.75 µCi/ml), and a glass capillary 1 mm in diameter was inserted inside. The tip of the capillary (red circle) was filled with 0.5 µl EO (1 mg/ml) and 0.5 µl ^{18}F-FDG (4.17 µCi/ml). (b) REFI showed clear contrast between the capillary tip and its surroundings (red arrow). (c) PET did not show clear contrast (black arrows).

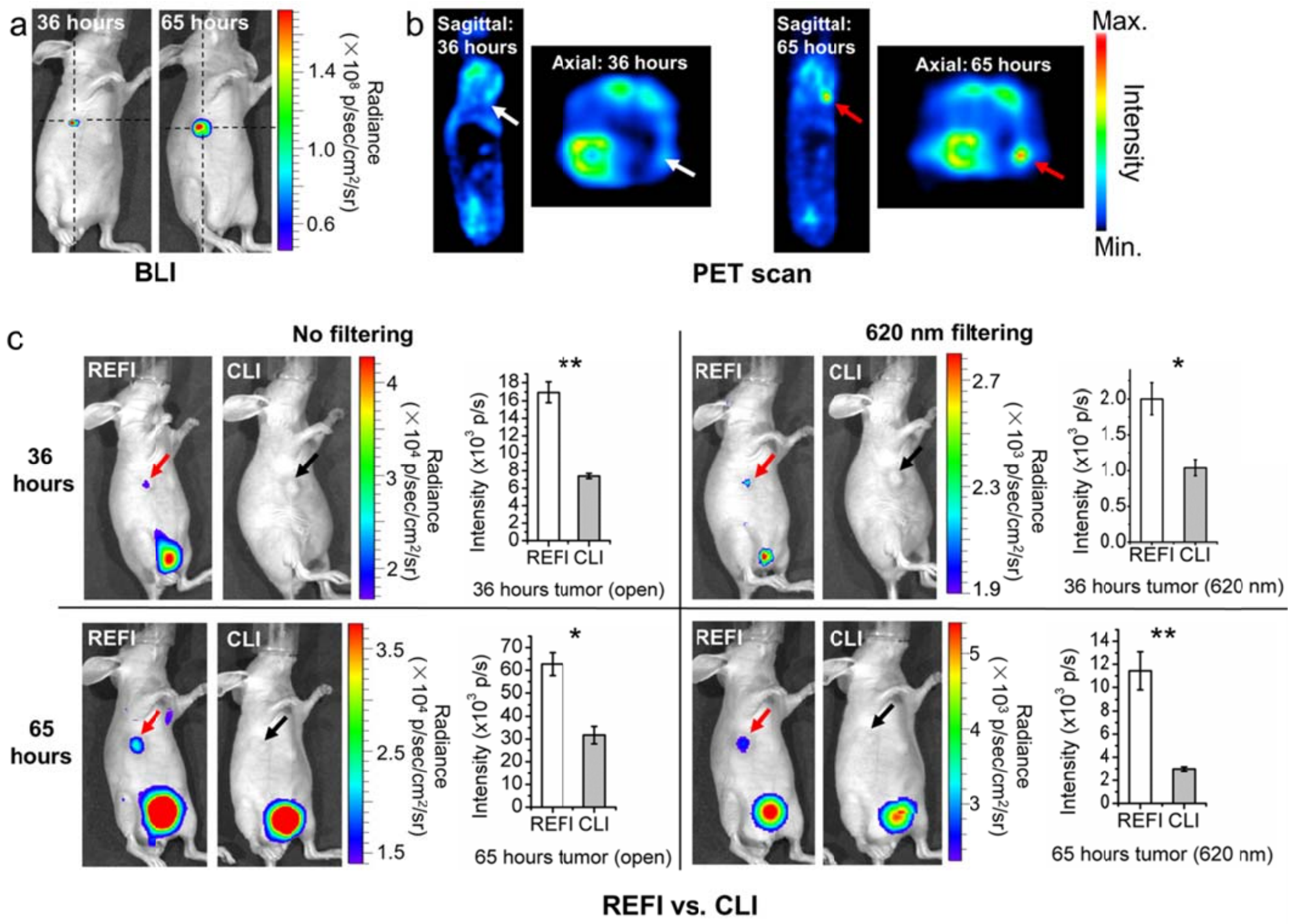

Supplementary Figure 5. *In vivo* multimodality comparison for early tumor detection. (a) BLI confirms the location of the tumor. The tumor size was 2.4 ± 0.3 mm at 36 hours' time point and 5.8 ± 0.5 mm at 65 hours' time point. (b) PET failed to detect the tumor lesion at the 36 hours' time point (left two images with white arrows), but successfully detected the tumor at the 65 hours' time point (right two images with red arrows). (c) With no filtering and 620 nm filtering, CLI showed false negative detection (black arrows), but REFI shows true positive detection (red arrows) of the tumor lesion, despite the time point being at 36 hours or 65 hours.

Supplementary Tables

Supplementary Table 1. The comparison of optical imaging techniques for both *in vitro* and *in vivo* phantom studies.

Phantoms	Signal-to-background ratio Mean ± SD			No filtering: intensity REFI *vs*. CLI Mean ± SD ($\times 10^5$ p/s)			620 nm filtered: intensity REFI *vs*. CLI mean ± SD ($\times 10^5$ p/s)		
	REFI	FMI	P	REFI	CLI	P	REFI	CLI	P
In vitro	73.3 ± 15.0	7.6 ± 0.8	<0.001	311.5 ± 33.5	83.3 ± 9.1	<0.001	130.0 ± 19.13	5.1 ± 0.9	<0.001
In vivo	71.2 ± 24.0	1.4 ± 0.3	<0.01	23.7 ± 3.8	16.6 ± 1.5	<0.05	1.6 ± 0.3	0.6 ± 0.26	<0.001

The signal-to-background ratio was defined as: (signal intensity-background)/background.

Supplementary Table 2. The comparison of optical imaging techniques for *in vivo* 4T1-luc2 tumor mouse model studies.

Mouse models		No filtering: intensity REFI *vs*. CLI Mean ± SD ($\times 10^4$ p/s)			620 nm filtered: intensity REFI *vs*. CLI mean ± SD ($\times 10^4$ p/s)			Signal-to-background ratio Mean ± SD			
		**REFI**	**CLI**	**P**	**REFI**	**CLI**	**P**	**REFI**	**FMI QD620**	**FMI RJ2-DG750**	**P**
**Dual tumor**	**Upper tumor**	8.8 ± 0.7	4.8 ± 0.1	<0.01	1.6 ± 0.1	0.6 ± 0.1	<0.001	2.7 ± 0.6	0.6 ± 0.02	N/A	<0.05
	**Lower tumor**	10.7 ± 0.2	7.5 ± 0.2	<0.01	1.7 ± 0.1	0.9 ± 0.2	<0.01	3.1 ± 0.6	0.4 ± 0.05	N/A	<0.05
**Small tumor**		3.9 ± 0.4	1.8 ± 0.5	<0.01	0.67 ± 0.01	0.30 ± 0.03	<0.01	4.4 ± 0.2	0.6 ± 0.1	1.5 ± 0.6	<0.05

The signal-to-background ratios of REFI were calculated from images after 620 nm filtering.
